# *Cave canem*: urine is not urine in corona times

**DOI:** 10.2217/fon-2020-0689

**Published:** 2020-08

**Authors:** Kunz Yannic, Horninger Wolfgang, Pinggera Germar-Michael

**Affiliations:** ^1^Department of Urology, Medical University Innsbruck, Anichstrasse 35, A-6020 Innsbruck, Austria

Dear Editor,

We read and appreciated the article “Implications of COVID-19 on urological laparoscopic surgery” by Condon *et al.* published on 9 June 2020 with great interest [1]. We wish to thank the authors for offering guidance in these difficult times of a SARS-CoV-2 pandemic. A comprehensive overview on surgical risk of infection with SARS-CoV-2 is given, as well as information on the reliability of current testing. While providing interesting information, especially on laparoscopic surgery, but also endourology, it is stated that SARS-CoV-2 has not been isolated in the urine thus far. Although positive respiratory, blood and feces specimens are correctly described in the article, the findings of detectable SARS-CoV-2 in the urine is still on debate and ongoing studies are providing further evidence. Therefore, we wish to emphasize that current evidence suggests a possible infectious hazard within the urine.

Since the outbreak of the severe acute respiratory syndrome coronavirus due to a enveloped, positive-sense, single-stranded RNA virus (SARS-CoV-1) in Asia in 2003, two important findings have been shown. First, it was demonstrated that SARS-CoV-1 enters different human cells by binding mainly to (ACE2) [2]. Second, in several investigations, the presence of this coronavirus could be detected not only in the epithelial cells within the lungs [3] and the feces but also in the urine. Chan *et al.* investigated the detection of SARS-CoV in 386 patients with serologically confirmed SARS-CoV infection, which was not detectable in feces or urine specimens until days 5 and 7 of the illness, respectively [4]. Interestingly, the specific virus detection increased in the following measurements and was peaking at approximately day 11 of the illness. Finally, they discovered that viral RNA gradually decreased from day 16 onward but was still detectable until 30 days of illness (up to day 7, 0% CoV+; days 7 and 8 under 10% CoV+; days 11 and 12, >40 to <50% CoV+; after day 30, approximately 5% CoV+) [4]. In the study from Peiris *et al.*, the detection rate for SARS-CoV in urine specimens by real-time (RT)-PCR was positive in 31 out of 74 investigated cases (41.9%) on day 14 [5]. Similar results were published by the SARS Study Group with around 30% positive urine specimens collected on days 10–15 after the onset of symptoms in 111 patients, with a pretty high viral load by quantitative RT-PCR (RT-qPCR) of 4.4 log10 copies/ml compared with 2.7 log10 copies/ml in the positive serum, respectively [6]. Nevertheless, viral urine cultures were positive for SARS-CoV in only 1 out of 20 performed measurements. Again, the SARS Study Group showed a detection rate of 26/177 (14.7%) in urine specimens of 177 SARS antibody-positive patients [6].

However, the current situation for SARS-CoV-2-related infectious disease is slightly different and not fully understood for now. The size of the actual SARS-CoV-2 genome is 29.9 kb, whereas the genomes of SARS-CoV and MERS-CoV are 27.9 kb and 30.1 kb, respectively and all of them are considered within the group of the betacoronavirus (the genome sequence of SARS-CoV-2 was first released on Virological.org) [7].

Meanwhile, it is clearly demonstrated that even SARS-CoV-2, which is genetically similar to the coronavirus strain SARS-CoV-1 virus, has likewise his primary human receptor by ACE2 [8,9], which was first identified in 2003 [10]. In the biolayer interferometry study, it was shown recently that SARS-CoV-2 has a similar affinity to ACE2, like SARS-CoV-1 [11]. Even though there is no doubt that the lung is the most severely injured organ by SARS-CoV-2 infection, SARS-CoV-2 can harm many other organs, such as the heart, liver, kidney, brain and intestines. This is always in conjunction with the widespread presence of the corresponding docking side in these organs [12–14]. Indeed, ACE2 has been identified as the SARS-CoV-2 receptor widely present in the human kidney and thus these findings of organ tropism are of high importance in understanding virus elimination, disease progression and fatality rate [15,16]. The ACE2 expressions as membrane-bound proteins are found mainly in the brush border of the proximal tubular units and, to a lesser extent, the podocytes, but not in the glomerular, endothelial and mesangial cells. Moreover, the net surface expression of this ACE2 receptor has been shown to be altered in several clinical conditions, such as diabetes, arterial hypertension and heart disease [17]. Once the kidneys’ morphological structures are damaged after SARS-CoV-2 infection, this might lead to acute kidney failure (AKI) [18,19]. By Kaplan–Meier analysis, it was demonstrated that renal failure had a greater risk for in-hospital mortality, and finally, Cox regression models confirmed AKI as an independent risk factor for predicting in-hospital patients’ mortality [10]. Therefore, it is conceivable that particles or complete SARS-CoV-2 virus might be detectable in the urine. It is possible that urinary excretion is dependent on the infection cycle or severity of tissue damage.

Thus, to assess and figure out any renal damage by SARS-CoV-2, a systematic, prospective investigation with an autopsy registry was initiated to facilitate COVID-19 research [20].

As stated in our recent review [21], three work groups isolated viral nucleic acid in the urine in various patients. This seems to be especially delicate since urine specimens remain positive even after a negative pharyngeal swab [22–24]. Still, more recent studies underline these findings. The Chinese work group of Ling *et al.* recently published a positive urine detection rate of 6.9% (4/58) [22]. This is supported by Zhang *et al.*, who observed a detection rate of 8.7% (2/23) [25] in one collective and 1.5% (1/67) in another [26]. Wang *et al.* investigated 48 patients with confirmed COVID-19 and proofed SARS-CoV-2 RNA in 6.25% (3/48) of cases [23]. Similarly, Peng *et al.* showed SARS-CoV-2 in one patient from a small positively tested collective, 11.1% (1/9) [24]. More recently, the Korean group by Kim *et al*. tested two patients positive in the urine in a bigger collective, 0.8% (2/247) [27] and by Sun *et al.* in a well-characterized patient case [28].

According to these six studies, roughly 3% of the tested patients showed positive results in the urine (13/452), posing a possible hazard for their caretakers. Therefore, disease transmission risk during urologic interventions by urine should not be completely neglected; especially urologists should not be careless in this field. Nevertheless, the transmission pathways of SARS-CoV-2 are not yet completely understood, the viral load might be only of transient character and the actual infection rate by SARS-CoV-2 via positive urine specimens remains to be investigated. In addition, the study designs are very different. Especially sample time differs vastly between the protocols, possibly explaining the different outcomes. Considering that SARS-CoV-2 could sometimes not be detected within the same patient at different measurements, there is implication for potentially increased unknown numbers.

On the contrary, we have to appreciate that there are several other studies not in line with the preceding findings. The following authors did not find any positive urine samples with any evidence of SARS-CoV-2: Kujawski *et al.* (n = 0/10) [29], Wolfel *et al.* (urine: n = 0/27 [0%] CoV+ samples) [30], Cai *et al.* (urine: n = 0/6 [0%] CoV+ [31], Chan *et al.* (urine: n = 0/3 [0%] patients CoV+ [32], Lo *et al.* (urine: n = 0/49 [0%] CoV+ [33] and Lescure *et al.* (urine: n = 0/5 [0%] CoV+ patients [34].)

Summarizing the mentioned data, it appears that these inherent study conflicts are explained by low sample analysis, unclear methodological quality of the studies, temperature and needed time of sample assessment, as well as time of sample investigation after disease onset.

Irrespective of these diverging results on urine contamination, it should be at least recognized that several study groups found SARS-CoV-2 viral load in the urine. Furthermore, SARS-CoV-2 was even detected in wastewater by RT-PCR [35–37]. In addition, we wish to present a Chinese study with 15% positive findings of SARS-CoV-2 in semen analysis as well, as this might concern urologists in their daily routine [38,39].

The safety of healthcare workers should be the primary concern and guideline to the treatment of COVID-19 patients. This is even more relevant in tandem with the newly published data on COVID-19 cases among healthcare workers [40]. We would like to emphasize that we strongly agree with the original authors in this instance.

Therefore, in accordance with the positive findings of SARS-CoV-2 in the urine mentioned previously, we recommend, in unison with the European Association of Urology guidelines on COVID-19 [41], special precautions even in endourology. The reassurance given by the original authors (Condon *et al.* [1]) at least for endourologists cannot be approved by us. Therefore, personal protective equipdment with FFP-2 masks and face shields should be utilized. Furthermore, closed suction devices for urine drainage seem reasonable. These recommendations are also in accordance with the newly published guidelines by strengthening all measures for protection of occupational health, safety and security of health workers by the WHO (WHO/2019-nCoV/HCF operations/2020.1) or others. However, especially oncologic endourologic interventions should not be postponed indefinitely in SARS-CoV-2 patients, when in fact net virus transmission by infected urine has not been described to date.
